# Phylogenomics reveals viral sources, transmission, and potential superinfection in early-stage COVID-19 patients in Ontario, Canada

**DOI:** 10.1038/s41598-021-83355-1

**Published:** 2021-02-12

**Authors:** Calvin P. Sjaarda, Nazneen Rustom, Gerald A. Evans, David Huang, Santiago Perez-Patrigeon, Melissa L. Hudson, Henry Wong, Zhengxin Sun, T. Hugh Guan, Muhammad Ayub, Claudio N. Soares, Robert I. Colautti, Prameet M. Sheth

**Affiliations:** 1 Queen’s Genomics Lab At Ongwanada (QGLO), Ongwanada Resource Center, Kingston, ON K7M8A6 Canada; 2 Department of Psychiatry, Queen’s University, Kingston, ON K7L3N6 Canada; 3 Centre for Neuroscience Studies, Queen’s University, Kingston, ON K7L3N6 Canada; 4 Division of Infectious Diseases, Department of Medicine, Queen’s University, Kingston, ON Canada; 5 Department of Pathology and Molecular Medicine, Queen’s University, Kingston, ON Canada; 6 Department of Biomedical and Molecular Sciences, Queen’s University, Kingston, ON Canada; 7 Biology Department, Queen’s University, Kingston, ON Canada; 8 Division of Microbiology, Kingston Health Sciences Center, Kingston, ON Canada; 9 Department of Family Medicine, Queen’s University, Kingston, ON Canada; 10 Gastrointestinal Disease Research Unit, Kingston Health Sciences Center, Kingston, ON Canada

**Keywords:** Evolutionary biology, Next-generation sequencing, Genome evolution, Viral infection, Viral infection, Epidemiology

## Abstract

The emergence and rapid global spread of SARS-CoV-2 demonstrates the importance of infectious disease surveillance, particularly during the early stages. Viral genomes can provide key insights into transmission chains and pathogenicity. Nasopharyngeal swabs were obtained from thirty-two of the first SARS-CoV-2 positive cases (March 18–30) in Kingston Ontario, Canada. Viral genomes were sequenced using Ion Torrent (n = 24) and MinION (n = 27) sequencing platforms. SARS-CoV-2 genomes carried forty-six polymorphic sites including two missense and three synonymous variants in the spike protein gene. The D614G point mutation was the predominate viral strain in our cohort (92.6%). A heterozygous variant (C9994A) was detected by both sequencing platforms but filtered by the ARTIC network bioinformatic pipeline suggesting that heterozygous variants may be underreported in the SARS-CoV-2 literature. Phylogenetic analysis with 87,738 genomes in the GISAID database identified global origins and transmission events including multiple, international introductions as well as community spread. Reported travel history validated viral introduction and transmission inferred by phylogenetic analysis. Molecular epidemiology and evolutionary phylogenetics may complement contact tracing and help reconstruct transmission chains of emerging diseases. Earlier detection and screening in this way could improve the effectiveness of regional public health interventions to limit future pandemics.

## Introduction

The past two decades have seen the emergence of three novel betacoronaviruses that have been associated with outbreaks in the human population including Severe Acute Respiratory Syndrome CoronaVirus (SARS-CoV) in 2002^1^, Middle East Respiratory Syndrome (MERS-CoV) in 2012^2^, and SARS-CoV-2 in 2019^3,4^. All three coronaviruses appear to have originated from bats and likely were transmitted to humans by zoonotic transmission possibly through an intermediate vertebrate vector^5,6^.

COrona VIrus Disease-2019 (COVID-19) is the infectious disease caused by the SARS-CoV-2. The first confirmed case dates to December 8, 2019 in a patient from Wuhan City in the Hubei province of China^7^. The virus quickly spread through Wuhan and neighbouring parts of Hubei province despite rapid and aggressive public health interventions^8^. The following months led to global spread of the virus and was officially classified as a pandemic by the WHO on March 11, 2020. The spread of SARS-CoV-2 has had devastating consequence to human health with over 40 million documented cases and 1.1 million deaths as of October 18, 2020 including 198,148 confirmed cases and 9760 deaths in Canada (WHO Weekly Epidemiological Update October 20).

SARS-CoV-2 is a spherical, enveloped particle, positive-sense, single stranded RNA genome that is 29.9 kb in length^3,9^. Genome organization of SARS-CoV-2 has the characteristic gene order 5′- replicase ORF1ab, spike (S), envelope (E), membrane (M), and nucleocapsid (N)-3′^6^. Coronavirus S proteins bind the host receptor enabling viral entry to the cell and have demonstrated the highest sequence variability in the viral genome^10^. The S protein in both SARS-CoV and SARS-CoV-2 interact with the host’s angiotensin-converting enzyme 2 (ACE2)^11^; however, the spike protein in SARS-CoV-2 has ~ 10- to 20-fold higher binding affinity than SARS-CoV^12^.

The complete SARS-CoV-2 genome was published on Jan 5, 2020 from a patient in Wuhan, China^3^. The collaborative effort of public health and research teams worldwide have now published 161,370 SARS-CoV-2 genomes in GISAID (www.gisaid.org) (as of October 27, 2020). A dynamic nomenclature system for SARS-CoV-2 has been described to facilitate real-time epidemiology revealing links between global outbreaks that share similar viral genomes^13^. At the root of the SARS-CoV-2 phylogeny are two lineages denoted A and B^13^. Although viruses from lineage B were sequenced and published first^3,6,14^, lineage A is likely ancestral as it shares two distinguishing variants with the closest known bat viruses^13^. Further linage designations link new variants to geographically distinct populations^13^. Some of the early lineages have been assigned to geographical locations including A.1 in Washington State, USA outbreak, B.1 in the Italian outbreak, then other parts of Europe and the world, and B.1.1 being the major European lineage which was spread throughout the world^13^. However, many of the major lineages are now present in most countries and recapitulate the global diversity of SARS-CoV-2 indicating that most local epidemics were seeded by a large number of independent introductions of the virus^15^.

Databases containing tens of thousands of SARS-CoV-2 genomes provide an unprecedented opportunity to reconstruct the establishment and spread of the virus in specific locales. Using SARS-CoV-2 genome sequencing data generated by ThermoFisher Scientific’s Ion Torrent and Oxford Nanopore Technology’s MinION next generation sequencing platforms, we traced the introduction and spread of the virus in some of first cases of COVID-19 in the eastern region of the province of Ontario, Canada. This knowledge may improve the effectiveness of public health interventions to prevent future pandemics.

## Materials and methods

### Sample collection

Nasopharyngeal (NP) swabs were collected in viral transport media from symptomatic patients being tested for SARS-CoV-2 at Kingston Health Sciences Center (KHSC) and the surrounding hospitals. Extraction of total RNA from viral transport media was performed using the Maxwell RSC Whole blood RNA/DNA kit (Promega Corporation, Madison, WI) on the Maxwell RSC 16 automated nucleic acid extractor. Each sample was tested for the presence of SARS-CoV-2 using a laboratory developed multiplex real-time PCR assay targeting the Envelope (E) and RNA dependent RNA polymerase (RdRp) genes^16^ on the ViiA7 Real-Time PCR System.

Biological samples and demographic data were collected from patients within the circle of care resulting from clinical testing for SARS-CoV-2. Samples were anonymized and de-identified so researchers were blind to the identity of the patients. Only secondary non-identifying data including age, biological sex, and travel history were provided. Under Ontario’s Personal Health Information Protection Act, all patients had the right to withhold or withdraw their consent for the use, access or disclosure of their Personal Health Information, and patients are not disadvantaged if they refuse to participate. The requirement for written informed consent was waived by the Research Ethics Board since we used anonymized and deidentified samples provided for clinical testing. All experimental protocols were approved by and conducted in accordance with the Queen’s University Health Sciences and Affiliated Teaching Hospitals Research Ethics Board (PSIY-676–20).

### SARS-CoV-2 genome sequencing via ion torrent

The extracted nucleic acids from COVID-19 positive cases were anonymized and shared with Queen’s Genomics Lab at Ongwanada (Q-GLO). RNA from each sample (5 μl) was reverse transcribed to complimentary DNA using the SuperScript VILO cDNA Synthesis Kit on a SimpliAmp Thermal Cycler. Libraries were constructed manually using the Ion AmpliSeq SARS-CoV-2 Research Panel, Ion Xpress Barcodes, and the Ion AmpliSeq Library Kit Plus following the manufacturer’s recommendations including amplification cycles based on viral load. Templating and chip loading were performed on the Ion Chef system using the Ion 510 & Ion 520 & Ion 530 Kit-Chef. Up to sixteen samples were multiplexed on an Ion 530 chip and sequenced using the Ion GeneStudio S5 Plus Semiconductor Sequencer (Supplementary Table 1). Preliminary analysis was performed on a Torrent Suite Server and using custom plug-ins created by ThermoFisher Scientific specifically for the Ion Ampliseq SARS CoV-2 panel including AssemblerTrinity for genome-guided assembly of the viral genome, IRMAreport to build a consensus sequence, and COVID19AnnotateSnpEff to annotate variants. VCF files were filtered to remove variants with quality score (-10logP) less than 400 (Supplementary Table 2).

### SARS-CoV-2 genome sequencing via MinION

Validation of SARS-CoV-2 genomes and variant lists generated by the Ion Torrent sequencing data was performed by parallel, independent sequencing on the MinION sequencing platform. Anonymized RNA samples were sent to the Queen’s Biology High Throughput Sequencing Core facility (Bio-HTS) where samples were reverse transcribed to cDNA using random hexamer primers and PCR amplified using a set of 109 primer pairs covering the whole viral genome (ARTIC Network amplicon sequencing protocol V2, with primers V3). Libraries were prepared from DNA with the Ligation Sequencing Kit (Oxford Nanopore Technologies) and sequenced on a FLO-MIN111 (R10.3) flow cell (Supplementary Table 1). Base calling and demultiplexing was performed in real time using MinKNOW v2.0. The assembly was performed in two steps (using default parameters) following the ARTIC Network bioinformatics protocol (https://artic.network/ncov-2019/ncov2019-bioinformatics-sop.html). The gupplyplex script was used for quality control and filtering of reads (fragments of 400 to 700 bp) followed by assembly with the MinION pipeline using the Wuhan-Hu-1 reference (GenBank accession number MN908947.3) (Supplementary Table 2).

### Chart review

Demographic data and travel history were extracted from a review of laboratory requisitions, hospital charts, and public health case investigation charts. Assessment of linked cases were based off public health case investigation charts.

### Phylogenetic analysis

A custom data analysis pipeline using molecular phylogenetics was developed to reconstruct infection origins and spread (https://github.com/ColauttiLab/SARS-CoV_Phylogenomics/commit/a691eecf4711f258ce3799ee3f30b0fb4c246024) (Supplementary Fig. 1). The pipeline follows the Nextstrain analysis^17^ but includes additional steps to remove genome sequences from divergent lineages not informative for reconstructing transmission. Non-informative sequences were filtered out by a pairwise comparison of each patient sample with each genome available in the GISAID database as of September 11, 2020. References sequences sharing the same multi-locus variants were retained from the database. Accessions containing the same set of variants were then grouped to tally origins (Supplementary Table 3). An ancestral reference sequence from Wuhan (Wuhan/WH04/2020) was then added to root the phylogenetic tree along with representative samples for each major lineage (Supplementary Table 4). A phylogenetic analysis of the 27 patient samples and 15 matching non-redundant reference sequences was estimated by maximum likelihood phylogenetic reconstruction using the ape (v5.3) and phangorn (v2.5.5) packages in R with 1,000 bootstrap iterations. All 23 available nucleotide substitution models were tested and model parameters were optimized using the *optim.pml* function. The ggtree (v2.0.1) and ggplot2 (v3.2.1) packages in R were used to generate final visualizations. Transmission inferences from the phylogenetic analysis assume that new mutations accrue at an average rate of 1 base pair (bp) every 7 to 21 days. This is based on an average of 24.225 bp substitutions per year in the Nextstrain analysis^17^ of the GISAID^18,19^ database. Genetic saturation was ruled out as only 0.1% (50 of 29,811 bp) of the genome was polymorphic and lineages had high bootstrap support (> 90%).

### Data availability

The consensus sequence for each sample was submitted to GISAID (https://www.gisaid.org/) under the accession IDs provided in Table 1.

**Table 1:** Summary of patient demographic data of first COVID-19 cases in the eastern region of the province of Ontario, Canada.

Sample ID	Age	Sex	Sample collection date	GISAID accession number	Lineage^a^	Countries with high lineage prevalence	Reported travel history or contact with known case
1	70 s	F	Mar 18	EPI_ISL_459866	B.1.1 (*GR*)	UK, USA, Portugal	Europe—Portugal
2	40 s	M	Mar 22	EPI_ISL_459867	B.1 (*GH*)	USA, UK, Australia	North America—Barbados
4	50 s	F	Mar 24	EPI_ISL_459868	B.1 (*GH*)	USA, UK, Australia	Contact sample 16
10	20 s	F	Mar 26	EPI_ISL_459869	B.1.5 (*G*)	UK, Spain, USA	Europe—Ireland
11	40 s	F	Mar 26	EPI_ISL_459871	B.1.114 (*GH*)	Canada, USA	Contact out of province
12	70 s	M	Mar 26	EPI_ISL_459872	B.1.1 (*GR*)	UK, USA, Portugal	Europe—Spain
16	60 s	M	Mar 26	EPI_ISL_459873	B.1 (*GH*)	USA, UK, Australia	Contact Sample 4
17	50 s	F	Mar 27	EPI_ISL_459874	B.1 (*GH*)	USA, UK, Australia	
18	20 s	M	Mar 27	EPI_ISL_459875	B.1.111 (*GH*)	Colombia, UK, USA	
19	70 s	M	Mar 27	EPI_ISL_459877	B.1.5 (*G*)	UK, Spain, USA	
21	80 s	M	Mar 27	EPI_ISL_459878	A.1 (*S*)	USA, Australia, Canada	
23	20 s	F	Mar 28	EPI_ISL_459879	A.1 (*S*)	USA, Australia, Canada	
24	50 s	M	Mar 28	EPI_ISL_459880	B.1 (*GH*)	USA, UK, Australia	Contact samples 25, 26
25	50 s	F	Mar 28	EPI_ISL_459881	B.1 (*GH*)	USA, UK, Australia	Contact samples 24, 26
26	20 s	M	Mar 28	EPI_ISL_459882	B.1 (*GH*)	USA, UK, Australia	Contact Samples 24, 25
29	60 s	M	Mar 28	EPI_ISL_459883	B.1 (*GH*)	USA, UK, Australia	No travel
30	80 s	F	Mar 28	EPI_ISL_459884	B.1 (*GH*)	USA, UK, Australia	
34	80 s	M	Mar 29	EPI_ISL_459885	B.1 (*GH*)	USA, UK, Australia	USA—Florida
35	70 s	M	Mar 30	EPI_ISL_459886	B.1 (*GH*)	USA, UK, Australia	
36	40 s	F	Mar 30	EPI_ISL_459887	B.1.3 (*GH*)	USA, Israel	
37	70 s	M	Mar 30	EPI_ISL_459888	B.1 (*GH*)	USA, UK, Australia	
38	20 s	F	Mar 30	EPI_ISL_459889	B.1 (*GH*)	USA, UK, Australia	No travel, contact
39	60 s	M	Mar 29	EPI_ISL_459890	B.1 (*GH*)	USA, UK, Australia	USA—Florida
40^b^	70 s	M	Mar 30	EPI_ISL_529029			
41	70 s	M	Mar 30	EPI_ISL_459891	B.1.2 (*GH*)	USA, Australia, Canada	USA—Arizona
42	60 s	F	Mar 30	EPI_ISL_459892	B.1 (*GH*)	USA, UK, Australia	No travel, contact
49	50 s	F	Mar 30	EPI_ISL_529030	B.1 (*GH*)	USA, UK, Australia	

Reported data includes anonymized sample ID, age (rounded to the decade), biological sex of the participant, date of sample collection, GISAID accession number for consensus sequence, PANGOLIN lineage (*GISAID Clade*)^a^, area of the world that have high prevalence of that viral lineage, and source of infection based on patient’s reported travel or contact history.

^a^Lineages assigned by Pangolin COVID-29 Lineage Assigner at https://pangolin.cog-uk.io/.

^b^Lineage not assigned due to low read coverage around position 14,408, a variant characteristic of B-lineage viruses.

## Results

Samples were obtained from thirty-two COVID-19 patients and sequenced by two independent laboratories, one using the Ion Torrent sequencing platform and the other using the MinION sequencing platform. Patient demographics including age and biological sex are shown in Table 1. The Ion Torrent platform successfully sequenced 24 of 32 samples with a mean of 1.3 million reads generating a mean of 8,279 times coverage (Supplementary Table 1). Samples 19 and 21 had the lowest uniformity in coverage which resulted in several gaps in the consensus sequence. The MinION sequencing platform successfully sequenced 27 of 32 samples with a mean of 145,000 reads generating a mean of 1780 times coverage. Samples 21 and 40 had the lowest coverage, which resulted in several gaps in the consensus sequence. The five samples that were unsuccessfully sequenced had very low viral load indicated by a high C_T_ value (Supplementary Table 1).

**Figure 1:**
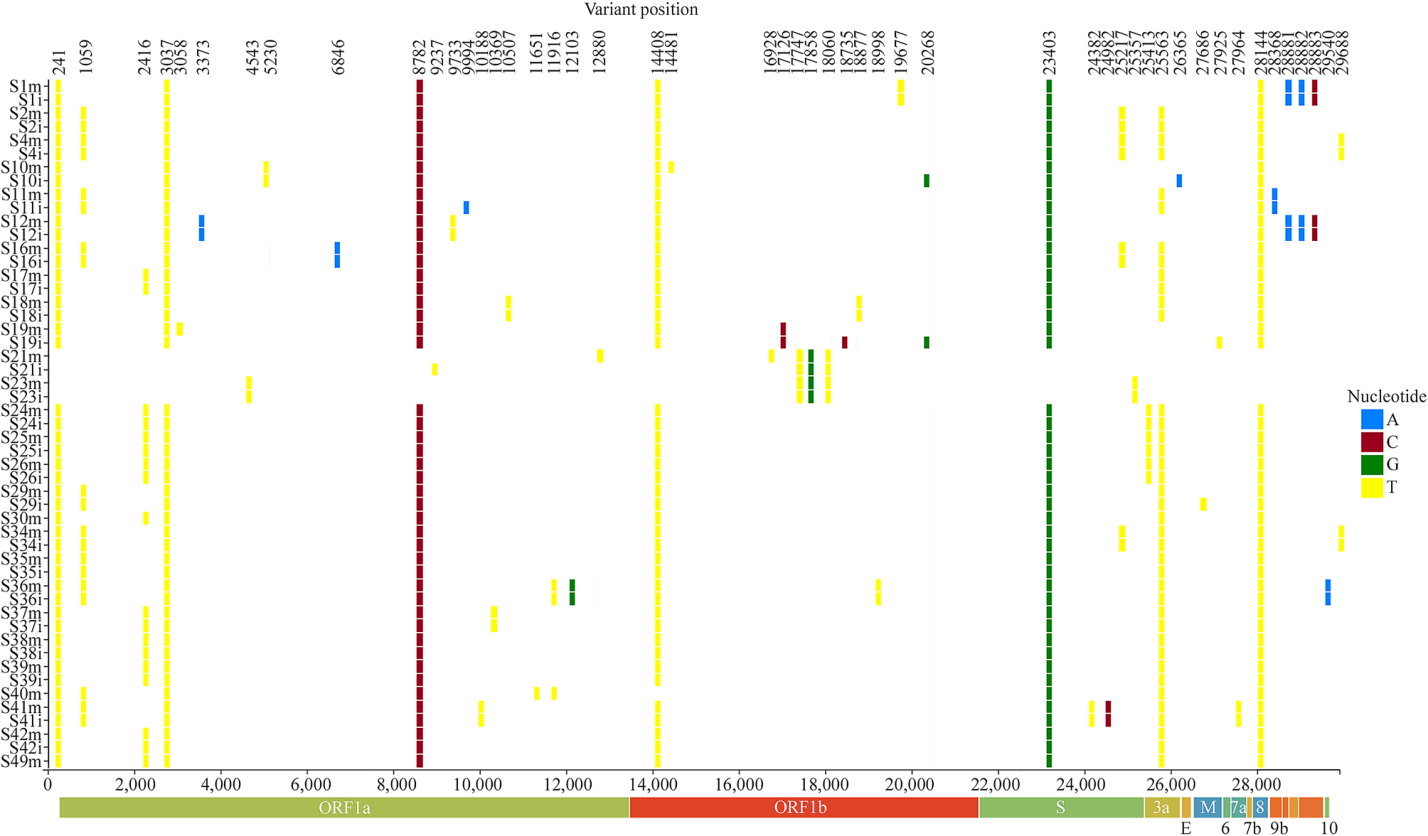
Distribution of polymorphisms in 27 SARS-CoV-2 genome sequences isolated from the early cases of COVID-19 in eastern Ontario. Viral genome sequencing identified forty-six polymorphic sites in twenty-seven viral genomes. S_m refers to the sample sequenced on the MinION and S_i refers to the sample sequenced on the Ion Torrent next generation sequencing platform.

The twenty-seven SARS-CoV-2 genomes carried a total of forty-six variants (Fig. 1; Supplementary Table 2). Most viral genomes contained between six to eight variants with a maximum of 12 variants in Sample 36 (S36). The most common nucleotide substitution is a cysteine to thymine transition (26/46 variants), followed by guanine to thymine transversion (5/46 variants) and adenine to guanine transition (4/46) (Supplementary Table 2). One heterozygous variant in the orf1ab gene of S11 (C9994A) was confirmed on both sequencing platforms, with no consistent evidence of heterozygous sequences in any of the other samples (Supplementary Table 2). There are two missense variants (A23403G and G25217T) and three synonymous variants (C24382T, T24982C, C25357T) in the gene encoding the S protein (Fig. 1; Supplementary Table 2).

Most variants were supported by both the Ion Torrent and MinION sequencing platforms (Supplementary Fig. 2). Three variants were uniquely called by the Ion Torrent sequencing platform (20268, 27686, and 27925), but there was not enough coverage in these areas in the MinION sequencing data to call these variants. On the other hand, four variants called by the MinION platform (3058, 3061, 12880, and 16928) were contradicted consensus sequences from the Ion Torrent dataset. A variant at position 9994 in sample S11 was the only heterozygous variant called in both sequencing methods, but the default MinION pipeline filtered out this variant (Supplementary Fig. 2 and Supplementary Fig. 3).

**Figure 2:**
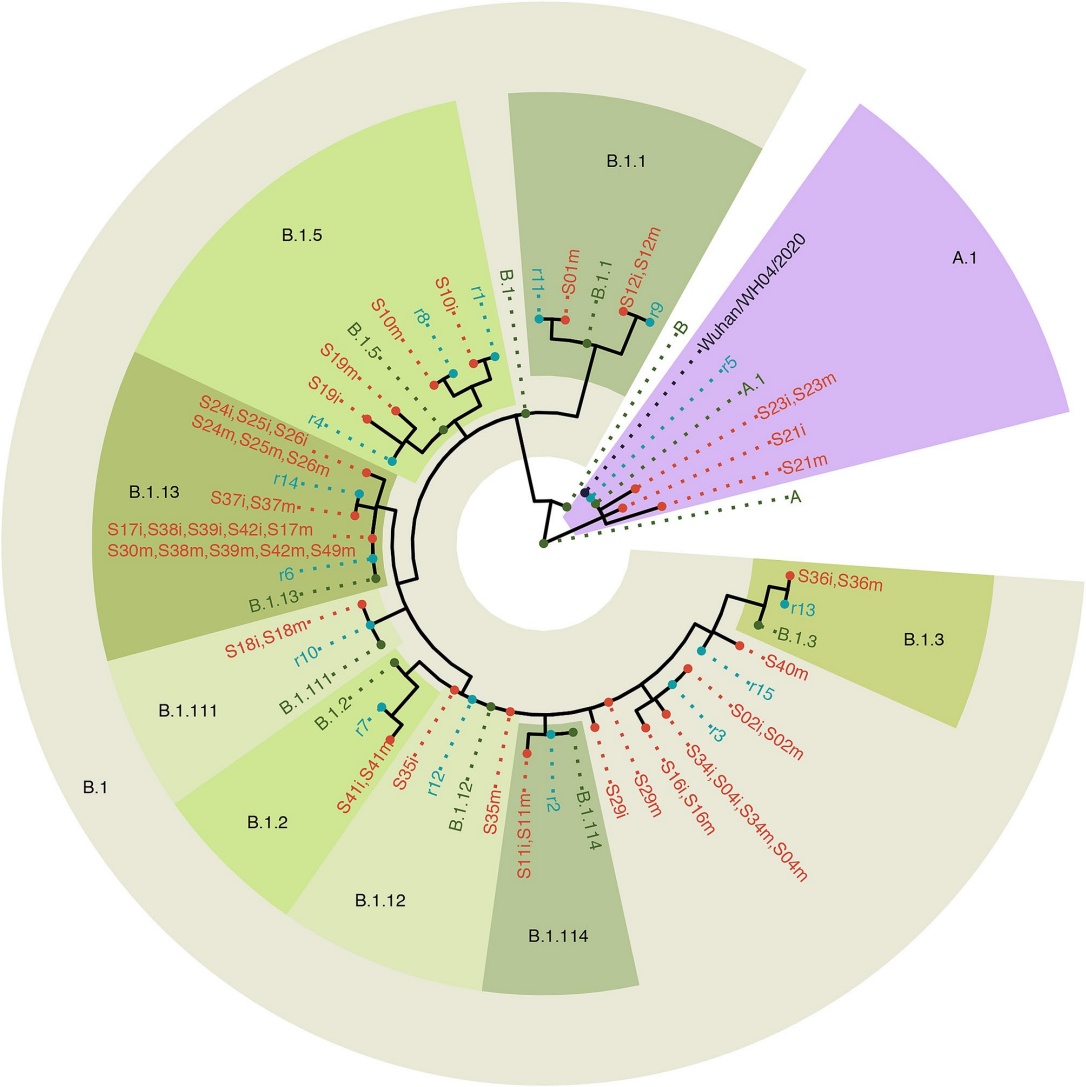
Nextstrain phylogenetic tree of local cases of SARS-CoV-2 and the most similar reference sequences in the GISAID database. Phylogenetic analysis suggests that S1, S10, S12, and S19 are similar to reference sequences that are predominately European. S21 and S23 are A.1 lineage viruses similar to reference sequences from the USA. The other samples are composed of B.1 and B.1 derived lineages and share genomes with reference sequences described predominantly in the USA. S_m refers to the sample sequenced on the MinION and S_i refers to the sample sequenced on the Ion Torrent next generation sequencing platforms; r_ refers to reference genome (Supplementary Table 3).

We used a phylogenomic analysis to reconstruct global origins and transmission events involving the 27 patient samples. The full analysis pipeline is available online (see GitHub link in methods) and outlined in Supplementary Fig. 1. Each of the 27 sequenced genomes was aligned and compared to each of 87,738 complete SARS CoV-2 genomes (of 96,295 submissions) made to the GISAID reference database as of September 11, 2020 (called reference sequences). Reference sequences were excluded from further analysis if they did not share polymorphisms with patient sequences, resulting in an informative reference set of 10,600 of the initial 87,738 genomes. Genomes representing each of the major evolutionary lineages^13^ were then added to the analysis to reconstruct origins (Supplementary Table 3). Samples S21 and S23 belong to the A.1 lineage of SARS-CoV-2 (Table 1) but differ by one to three bp from the closest reference genomes in the GISAID database (Fig. 2). Samples in the A.1 lineage are most common in the USA, Canada, and Australia (Table 1) and reference genome 5 consists almost exclusively of samples from the USA and Canada (Supplementary Table 3) suggesting transmission within Canada or from the USA. However, there was no patient travel history information to validate this prediction. The remaining patient samples are all derived from the B.1 lineage. S1 and S12 belong to the B.1.1 lineage of SARS-CoV-2 which is most prevalent in the UK, USA, and Portugal (Table 1) while S10 and S19 belonging to the B.1.5 lineage which is most prevalent in the UK, Spain, and USA (Table 1) (Fig. 2). These samples matched reference genomes 1, 4, 8, 9, and 11 in the GISAID database (Supplementary Table 3) and these come primarily from the UK and Spain, suggesting that these four samples represent four distinct transmission events from Europe into Canada. There is no patient travel history information for S19 but reported travel history supports European origin for S1 (travel to Europe–Portugal), S10 (travel to Europe–Ireland), and S12 (travel to Europe–Spain) (Table 1). A cluster of samples in the B.1.13 lineage suggests a shared source of infection or community transfer related to reference sequence 6, which is represented by dozens of samples primarily in the USA but also in Europe, Australia, Asia and the Middle East (Supplementary Table 3). Samples in the B.1.2 lineage (S41), B.1.3 lineage (S36), B.1.111 lineage (S18) and B.1.114 lineage (S11) are likewise prevalent in the USA, suggesting that many of the samples on lineage B.1 may have been introduced from the USA. Reported travel history for several participants supports the USA origin for these samples including S34 (USA–Florida), S39 (USA–Florida) and S41 (USA–Arizona). Community transfer is supported by reported contact history for at least one of these clusters as S24, S25 and S26 did not travel and were in contact with each other.

## Discussion

Large databases of genomic data for human pathogens have stimulated the field of phylodynamics, an intersection of immunodynamics, epidemiology, and evolutionary biology, to understand infectious disease dynamics using pathogen phylogenies^20,21^. Phylogenetic analysis and molecular epidemiology have been used to describe the introduction and transmission of SARS-CoV-2 during early phases of the pandemic in several regions including Italy^22,23^, the Netherlands^24,25^, Chile^26^, Northern California^27^, and New York City^28^. Here we report twenty-seven SARS-CoV-2 genomes from cases of COVID-19 within the eastern region of the province of Ontario, Canada. These samples included most of the first cases in the region and therefore infection was thought to have occurred during international travel or while in proximity to an individual who had recently travelled. Genome variation among these samples has important implications for the pathology and epidemiology of this disease, as discussed below.

A major concern with novel viruses is mutation rates and how novel mutations will affect virulence, vaccine development, and reinfection^29–33^. Of primary significance are mutations in the S protein because the spike protein defines viral host range and is often the target of neutralizing antibodies^34,35^. This project identified three unique mutations in the coding region of the S protein, however all three are predicted to be synonymous and likely will not affect viral virulence or epitopes (Supplementary Table 2: C24382T, T24982C, and C25357T). A fourth polymorphic site at A23403G is a well described mutation that results in a D614G substitution in the S protein (Fig. 1) and may represent a strain of SARS-CoV-2 with increased fitness^33^. This mutation is also embedded in an immunological epitope which elicited antibody production in patients during the 2003 SARS-CoV epidemic^30^, and may mediate antibody-dependent enhancement of infection^33^. Prior to March 1st 2020, the D614 mutation was dominate in most countries around the world, comprising ~ 90% of all global sequences^33^. However, a global transition from D614 to G614 has occurred since March 1^st^ with the G614 mutation representing 67% of all sequenced genomes by March 31 and 78% by May 18 observed first in Europe, then North America, then the rest of world^33^. The G614 variant is characteristic of all SARS-CoV-2 genomes in the B.1 and its descendant lineages^36^, and many countries that avoided a first wave of SARS-CoV-2 in January and February 2020 report SARS-CoV-2 genotypes that are almost exclusively the G614 variant^37^. As COVID-19 cases were first introduced in Eastern Ontario from Europe and the USA in mid-late March, it was not unexpected that the G614 mutation comprises 92.6% of cases in this study. A fifth polymorphic site (C25217T) was observed in four samples (S2, S4, S16, and S34,) and is a missense variant resulting in a glycine to cysteine substitution at the 1219th residue of the S protein. In silico modeling or functional validation studies may describe the impact of this mutation on the function of the transmembrane domain. We also identified one heterozygous variant, C9994A, coding for a missense variant in ORF1a in S11. This variant was called by both the Ion Torrent and MinION sequencing datasets, though the default ARTIC Network bioinformatics protocol filtered out this variant. Although COVID-19 patients exhibiting within-host diversity of multiple SARS-CoV-2 strains have been described^8,38^, this phenomenon may be underreported in the SARS-CoV-2 databases and literature if the default analysis pipeline is routinely removing heterozygous variants. Whether the variant is a superinfection or an emerging variant, detecting heterozygous variants empower contact tracing and mutation tracking efforts and should be further investigated in SARS-CoV-2 whole genome sequencing data.

Clustering of shared mutations identified two samples (S21 and S23) that belong to A.1 lineage characterized by two polymorphic sites at C8782T and T28144C^13^. The presence of three additional polymorphic sites at C17747T, A17858G, and C18060T exclusively present in North America^32^ provide support for the USA origin hypothesis described by our phylogenetic analysis. Within the other twenty-five samples sharing the D614G substitution in the S protein, we observe three distinct lineages. Two of these, lineage B.1.1 (S1 and S12) and B.1.5 (S10 and S19) appear European in origin. The third cluster includes the remaining sequences in B.1 (S2, S4, S16, S17, S24, S25, S26, S29, S30, S34, S35, S37, S38, S39, S42, S49), as well as sub-lineages B.1.2 (S41), B.1.3 (S36), B.1.111 (S18), and B.1.114 (S11), all of which appear to originate in the USA. Reported travel history supports our phylogenetic analysis for European origin for S1, S10 and S12 as S1 reported recent travel to Portugal, S10 travelled to Ireland, and S12 to Spain. Similarly, reported travel history supports North American origin for several samples as S34 and S39 reported recent travels to Florida, USA and S41 reported travel to Arizona, USA. The validation of phylogenetic origin by reported travel history suggest that viral genome sequencing and phylogenetic analysis can suggest potential sources of SARS-CoV-2 infection into Canada. Interestingly, we observed several clusters of samples that shared the same viral sequence indicating that these samples either had a shared source of infection or were a result of community transfer. For example, S24, S25 and S26 are identical suggesting a common transmission source. Chart review confirmed that patients S24, S25, and S26 did not travel outside of Canada and were in contact with each other, demonstrating an early example of community transfer in eastern Ontario. Patient samples S17, S30, S38, S39 and S42 also share the same viral strain but the connection between these individuals is unknown. Identification of the common thread among these patients (or other clusters of cases) could help to identify major sources/pathways of infection.

There were several limitations in using genomics to trace viral introduction and transmission of SARS-CoV-2. First, in the early stages of the pandemic, most countries were only screening and testing international travelers who displayed symptoms. This allowed asymptomatic carriers and community transmission to go undetected as sources of infection. As a result, gaps exist in the databases reporting SARS-CoV-2 genomes that may not include the original sources of infection. However, the rapid publication of genome sequences from around the world can help to offset this limitation by identifying geographical clusters and specific genomic variants that are shared across regions. The mutation rate of SARS-CoV-2 (~ 6 × 10^–4^ nucleotides/genome/year^15^) is fast enough to distinguish primary and secondary infections in the span of 1–2 weeks yet slow enough to clearly distinguish evolutionary origins in a phylogenetic analysis. A second limitation is a lack of detailed travel and interaction histories for patients due to differences in reporting and data collection among collection sites and agencies. Rigorous adherence to standardized data collection protocols, like WHO’s guidance for contact tracing in the context of COVID-19 coupled with genomics data as described here, may facilitate effective contact tracing that is required to break the chains of viral transmission. This information can help to validate inferences from phylogenetic analysis^27^. A final limitation is that sequencing SARS-CoV-2 in COVID-19 cases with low viral load was problematic due to lack of RNA input for library construction as seen by the number of excluded samples. However, this will become less limiting over time as high-throughput sequencing devices continue to improve in sensitivity and throughput.

In summary, this is the first description of SARS-CoV-2 genomes in COVID-19 positive cases in Canada. These are many of the first detected cases in eastern Ontario and infection was believed to be foreign in origin and the result of international travel or proximity to an individual who had travelled. This may be one of the first studies to sequence the same samples on multiple NGS platforms, allowing us to identify a potential superinfection or emerging viral variant in S11 and that the default ARTIC network bioinformatic protocol appears to be filtering heterozygous variants. Furthermore, the majority of viral strains introduced into Eastern Ontario in March 2020 already harboured the G614 mutation and may have facilitated the local spread of the virus as the mutation has been associated with enhanced viral fitness. Our phylogenetic analysis and contact tracing suggest that many of the infections originated from our geographical neighbour, the USA, but other sources of infection may include several countries in Europe. We also observed community transfer in cases that did not report travel out of the country. These results demonstrate how molecular epidemiology and evolutionary phylogenetics can help local health units to track origins and vectors of spread for emerging diseases like SARS-CoV-2. Earlier detection and screening and alternative modes for contact tracing may improve the effectiveness of regional public health interventions to prevent future pandemics.

## Supplementary Information

Supplementary Information
