# Supplementary material for: Phylogenomics reveals viral sources, transmission, and potential superinfection in early-stage COVID-19 patients in Ontario, Canada
Source: Sci Rep. 2021 Feb 12;11:3697. doi: 10.1038/s41598-021-83355-1 (PMC7881023; doi:10.1038/s41598-021-83355-1)
Supplement: Supplementary file 1 — Supplementary Information [file 41598_2021_83355_MOESM1_ESM.docx]

**Title**

Phylogenomics reveals viral sources, transmission, and potential superinfection in early-stage COVID-19 patients in Ontario, Canada

**Short title**

SARS-CoV-2 genomes from Canadian patients

**Authors**

Calvin P Sjaarda^1,2*^, Nazneen Rustom^2,3^, Gerald A Evans^4,5,6^, David Huang^7^, Santiago Perez-Patrigeon^4^, Melissa L Hudson^1,2^, Henry Wong^8^, Zhengxin Sun^7^, T Hugh Guan^9^, Muhammad Ayub^2,3^, Claudio N Soares^1,2^, Robert I Colautti^7†^, Prameet M Sheth^5,6,8,10†^

**Affiliations**

^1^ Queen's Genomics Lab at Ongwanada (QGLO), Ongwanada Resource Center, Kingston, Ontario, Canada, K7M8A6

^2^ Department of Psychiatry, Queen’s University, Kingston, Ontario, Canada, K7L3N6

^3^ Centre for Neuroscience Studies, Queen’s University, Kingston, Ontario Canada, K7L3N6

^4^ Department of Medicine, Division of Infectious Diseases, Queen’s University, Kingston, Ontario

^5^ Department of Pathology and Molecular Medicine, Queen’s University, Kingston, Ontario, Canada

^6^ Department of Biomedical & Molecular Sciences, Queen’s University, Kingston, Ontario

^7^ Biology Department, Queen’s University, Kingston, Ontario, Canada

^8^ Division of Microbiology, Kingston Health Sciences Center, Kingston, Ontario, Canada

^9^ Department of Family Medicine, Queen’s University, Kingston, Ontario, Canada

^10^ Gastrointestinal Disease Research Unit, Kingston Health Sciences Center, Kingston, Ontario.

†These authors contributed equally to this work

*Corresponding Author

Calvin P Sjaarda, PhD

Email: [calvin.sjaarda@queensu.ca](mailto:calvin.sjaarda@queensu.ca)

**Supplementary Table 1. Summary of SARS-CoV-2 viral load, sequencing reads, coverage, and variants called by the Ion Torrent and the Nanopore MinION sequencing platforms for each COVID-19 positive sample.** Several samples had insufficient number of mapped reads, coverage, or coverage uniformity which made variant calling and generation of a consensus sequences unachievable (shown by grey highlighting).

| Sample ID | C_T_ value | Ion Torrent Mapped reads | Ion Torrent Coverage | Ion Torrent Coverage uniformity | Ion Torrent Number of variants | MinION Mapped reads | MinION Coverage | MinION Number of variants |
| --- | --- | --- | --- | --- | --- | --- | --- | --- |
| 1 | 33 | 1,582,943 | 9,215 | 97.80% | 6 | 115,193 | 1,370.8 | 8 |
| 2 | 31 | 1,429,307 | 9,478 | 96.47% | 7 | 178,360 | 2,118.8 | 7 |
| 4 | 18 | 1,730,539 | 11,809 | 97.89% | 8 | 120,384 | 1,498.8 | 8 |
| 10 | 29 | 1,429,167 | 8,851 | 99.69% | 7 | 173,011 | 2,127.5 | 6 |
| 11 | 27 | 639,006 | 4,305 | 97.58% | 8 | 199,133 | 2,466.9 | 7 |
| 12 | 25 | 1,148,216 | 7,560 | 98.21% | 7 | 174,216 | 2,161.0 | 9 |
| 16 | 23 | 1,368,607 | 9,294 | 98.00% | 8 | 191,946 | 2,375.0 | 8 |
| 17 | 19 | 1,573,637 | 10,745 | 98.13% | 6 | 83,478 | 1,084.7 | 6 |
| 18 | 31 | 1,316,806 | 6,920 | 99.17% | 7 | 154,087 | 1,818.2 | 7 |
| 19 | 34 | 1,044,713 | 3,170 | 71.19% | 11 | 105,556 | 1,129.6 | 8 |
| 21 | 34 | 1,082,078 | 2,054 | 71.65% | 12 | 60,300 | 638.8 | 8 |
| 23 | 28 | 1,252,788 | 8,510 | 98.19% | 7 | 205,514 | 2,478.5 | 8 |
| 24 | 22 | 1,790,583 | 12,325 | 98.20% | 7 | 174,995 | 2,174.4 | 7 |
| 25 | 23 | 1,731,460 | 11,783 | 98.21% | 7 | 131,584 | 1,634.6 | 7 |
| 26 | 20 | 1,512,017 | 10,267 | 98.18% | 8 | 170,798 | 2,120.2 | 7 |
| 29 | 26 | 1,429,785 | 9,670 | 98.55% | 7 | 98,762 | 1,260.3 | 6 |
| 30 | 26 | NA | NA | NA | NA | 163,719 | 2,026.2 | 6 |
| 34 | 22 | 876,823 | 5,750 | 98.74% | 8 | 181,365 | 2,242.3 | 8 |
| 35 | 21 | 1,277,367 | 8,578 | 98.09% | 7 | 86,198 | 1,114.4 | 6 |
| 36 | 29 | 1,541,104 | 10,140 | 99.14% | 12 | 181,741 | 2,210.7 | 12 |
| 37 | 21 | 632,145 | 994 | 98.28% | 7 | 201,694 | 2,446.9 | 7 |
| 38 | 27 | 1,470,901 | 9,982 | 98.55% | 6 | 91,623 | 1,170.5 | 6 |
| 39 | 37 | 1,105,265 | 7,350 | 97.22% | 6 | 185,498 | 2,297.6 | 6 |
| 40 | 24 | 193,162 | 140.9 | 12.77% | 0 | 63,875 | 631.1 | 7 |
| 41 | 27 | 1,356,131 | 9,297 | 98.71% | 10 | 199,986 | 2,442.7 | 10 |
| 42 | 22 | 1,562,381 | 10,640 | 98.20% | 6 | 118,863 | 1,508.8 | 6 |
| 45 | 35 | 822,950 | 1,956 | 1.50% | 0 | 1,002 | 3.9 | 0 |
| 46 | 37 | 749,060 | 1,687 | 1.50% | 0 | NA | NA | NA |
| 47 | 36 | 760,725 | 1,829 | 1.50% | 1 | 7,778 | 6.1 | 0 |
| 48 | 36 | 829,589 | 1,955 | 1.55% | 0 | 7,266 | 4.9 | 0 |
| 49 | 20 | 570 | 2.404 | 78.71% | 0 | 119,122 | 1,510.8 | 6 |
| 50 | 37 | 861,289 | 763.6 | 1.55% | 0 | 14,162 | 6.6 | 0 |

**Supplementary Table 2. Forty-six variants were observed in twenty-seven SARS-CoV-2 viral genome sequences isolated from COVID-19 cases in Eastern Ontario, Canada.**

| Variant position | Genotype | Gene | HGVS_C | HGVS_P | Effect | # samples carrying variant | Ion Torrent quality score  (-10logP) | MinION quality score  (-10logP) |
| --- | --- | --- | --- | --- | --- | --- | --- | --- |
| 241 | Homozygous | orf1ab | c.-25C>T | . | upstream_gene_variant | 25 | 2979.64 | 2990.3 |
| 1059 | Homozygous | orf1ab | c.794C>T | p.Thr265Ile | missense_variant | 10 | 2696.78 | 3371.2 |
| 2416 | Homozygous | orf1ab | c.2151C>T | p.Tyr717Tyr | synonymous_variant | 10 | 2982.21 | 3103.8 |
| 3037 | Homozygous | orf1ab | c.2772C>T | p.Phe924Phe | synonymous_variant | 25 | 2957.67 | 3468.8 |
| ^a^ 3058 | Homozygous | orf1ab | c.2793A>T | p.Glu931Asp | missense_variant | 1 | NA | 757.9 |
| ^a^ 3061 | Homozygous | orf1ab | c.2796delinsA>AG | p.Glu933Glyfs*3 | frameshift_substitution | 1 | NA | 757.9 |
| 3373 | Homozygous | orf1ab | c.3108C>A | p.Asp1036Glu | missense_variant | 1 | 2977.9 | 4696.2 |
| 4543 | Homozygous | orf1ab | c.4278C>T | p.Thr1426Thr | synonymous_variant | 1 | 2979.58 | 2148.6 |
| 5230 | Homozygous | orf1ab | c.4965G>T | p.Lys1655Asn | missense_variant | 1 | 2981.78 | 245.2 |
| 6846 | Homozygous | orf1ab | c.6581T>A | p.Met2194Lys | missense_variant | 1 | 2982.2 | 613.7 |
| 8782 | Homozygous | orf1ab | c.8517C>T | p.Ser2839Ser | synonymous_variant | 2 | 2919.61 | 2653.6 |
| 9733 | Homozygous | orf1ab | c.9468C>T | p.Phe3156Phe | synonymous_variant | 1 | 2345.88 | 3610.9 |
| ^b^ 9994 | Heterozygous | orf1ab | c.9729C>A | p.Asn3243Lys | missense_variant | 2 | 747.545 | 405.5 |
| 10188 | Homozygous | orf1ab | c.9923C>T | p.Thr3308Ile | missense_variant | 1 | 2946.18 | 3129.1 |
| 10369 | Homozygous | orf1ab | c.10104C>T | p.Arg3368Arg | synonymous_variant | 1 | 2919.63 | 2113.5 |
| 10507 | Homozygous | orf1ab | c.10242C>T | p.Asn3414Asn | synonymous_variant | 1 | 2976.7 | 1814.1 |
| 11651 | Homozygous | orf1ab | c.11386C>T | p.Leu3796Phe | missense_variant | 1 | NA | 124.8 |
| 11916 | Homozygous | orf1ab | c.11651C>T | p.Ser3884Leu | missense_variant | 2 | 2912.26 | 4323.4 |
| 12103 | Homozygous | orf1ab | c.11838A>G | p.Ser3946Ser | synonymous_variant | 1 | 2948.9 | 2653.6 |
| ^a^ 12880 | Homozygous | orf1ab | c.12615C>T | p.Ile4205Ile | synonymous_variant | 1 | NA | 3568.4 |
| 14408 | Homozygous | orf1ab | c.14143C>T | p.Leu4715Leu | synonymous_variant | 24 | 2982.16 | 2763.6 |
| 14481 | Homozygous | orf1ab | c.14216C>T | p.Thr4739Ile | missense_variant | 1 | 1025.96 | 3587.6 |
| ^a^ 16928 | Homozygous | orf1ab | c.16663C>T | p.Thr5555Ile | missense_variant | 1 | NA | 930.2 |
| 17126 | Homozygous | orf1ab | c.16861T>C | p.Ser5621Pro | missense_variant | 1 | 2978.36 | 3061.5 |
| 17747 | Homozygous | orf1ab | c.17482C>T | p.Leu5828Leu | synonymous_variant | 2 | 2949.23 | 2457.4 |
| 17858 | Homozygous | orf1ab | c.17593A>G | p.Met5865Val | missense_variant | 2 | 2376.41 | 2223.3 |
| 18060 | Homozygous | orf1ab | c.17795C>T | p.Ser5932Phe | missense_variant | 2 | 2981.21 | 2464.0 |
| 18877 | Homozygous | orf1ab | c.18612C>T | p.Val6204Val | synonymous_variant | 1 | 2964.71 | 3978.8 |
| 18998 | Homozygous | orf1ab | c.18733C>T | p.His6245Tyr | missense_variant | 1 | 2948.89 | 2207.5 |
| 19677 | Homozygous | orf1ab | c.19412G>T | p.Arg6471Met | missense_variant | 1 | 2974.77 | 434.1 |
| ^c^ 20268 | Homozygous | orf1ab | c.20003A>G | p.Ter6668Trpext*? | stop_lost | 2 | 2919.39 | NA |
| 23403 | Homozygous | S | c.1841A>G | p.Asp614Gly | missense_variant | 25 | 2982.2 | 3792.8 |
| 24382 | Homozygous | S | c.2820C>T | p.Ser940Ser | synonymous_variant | 1 | 2948.9 | 1825.8 |
| 24982 | Homozygous | S | c.3420T>C | p.Pro1140Pro | synonymous_variant | 1 | 2870.14 | 1727.1 |
| 25217 | Homozygous | S | c.3655G>T | p.Gly1219Cys | missense_variant | 4 | 2982.21 | 4220.6 |
| 25357 | Homozygous | S | c.3795C>T | p.Leu1265Leu | synonymous_variant | 1 | 2952.1 | 2959.4 |
| 25413 | Homozygous | ORF3a | c.21C>T | p.Ile7Ile | synonymous_variant | 3 | 2975.89 | 3926.5 |
| 25563 | Homozygous | ORF3a | c.171G>T | p.Gln57His | missense_variant | 21 | 2982.21 | 4597.4 |
| ^c^ 27686 | Homozygous | ORF7a | c.293C>T | p.Ser98Phe | missense_variant | 1 | 2972.12 | NA |
| ^c^ 27925 | Homozygous | ORF8 | c.32C>T | p.Thr11Ile | missense_variant | 1 | 2699.81 | NA |
| 27964 | Homozygous | ORF8 | c.71C>T | p.Ser24Leu | missense_variant | 1 | 2964.9 | 1107.7 |
| 28144 | Homozygous | ORF8 | c.251T>C | p.Leu84Ser | missense_variant | 2 | 2982.08 | 3350.2 |
| 28368 | Homozygous | N | c.95G>A | p.Arg32His | missense_variant | 1 | 2982.21 | 2490.7 |
| 28881 | Homozygous | N | c.608_610delGGGinsAAC | p.ArgGly203LysArg | missense_variant | 2 | 2959.76 | 5307.9 |
| 29540 | Homozygous | ORF10 | c.-18G>A | . | upstream_gene_variant | 1 | 2919.65 | 780.5 |
| 29688 | Homozygous | S | c.*4304G>T | . | downstream_gene_variant | 2 | 2919.15 | 4624.2 |

^a^ Variants 3058, 3061, 12880, and 16928 were called by the MinION platform, but these variants were not supported by the Ion Torrent platform (see S1 Figure).

^b^ Variant 9994 was called by both sequencing platforms but filtered out by MinION (see S1 Figure and S2 Figure).

^c^ Variants 20268, 27686, and 27925 were called by the Ion Torrent platform, but there was no sequencing coverage in these areas from the MinION platform (see S1 Figure).

**Supplementary Table 3. Location of published SARS-CoV-2 sequences that share polymorphisms with viral genomes isolated for COVID-19 cases in the eastern region of the province of Ontario, Canada.**

| Reference genome ID | Region | N |
| --- | --- | --- |
| r1 | Other | 1 |
| r2 | Canada | 14 |
| r2 | Other | 8 |
| r3 | Canada | 11 |
| r3 | USA | 31 |
| r3 | Other | 6 |
| r4 | Scotland | 17 |
| r4 | Spain | 22 |
| r4 | Other | 12 |
| r5 | Canada | 35 |
| r5 | USA | 431 |
| r5 | Other | 6 |
| r6 | Australia | 36 |
| r6 | Canada | 42 |
| r6 | England | 46 |
| r6 | France | 42 |
| r6 | Israel | 28 |
| r6 | USA | 609 |
| r6 | Other | 55 |
| r7 | Australia | 43 |
| r7 | Canada | 11 |
| r7 | England | 11 |
| r7 | USA | 90 |
| r7 | Vietnam | 15 |
| r7 | Other | 34 |
| r8 | Other | 4 |
| r9 | Canada | 16 |
| r9 | Other | 17 |
| r10 | Canada | 29 |
| r10 | Colombia | 19 |
| r10 | USA | 32 |
| r10 | Other | 9 |
| r11 | Canada | 19 |
| r11 | England | 291 |
| r11 | Scotland | 18 |
| r11 | Spain | 22 |
| r11 | Wales | 22 |
| r11 | Other | 17 |
| r12 | Australia | 282 |
| r12 | Austria | 83 |
| r12 | Belgium | 45 |
| r12 | Canada | 144 |
| r12 | Chile | 13 |
| r12 | Colombia | 27 |
| r12 | Denmark | 284 |
| r12 | DRC | 10 |
| r12 | England | 442 |
| r12 | Finland | 51 |
| r12 | France | 155 |
| r12 | Germany | 48 |
| r12 | Iceland | 76 |
| r12 | Israel | 120 |
| r12 | Luxembourg | 35 |
| r12 | Netherlands | 53 |
| r12 | New Zealand | 10 |
| r12 | Northern Ireland | 11 |
| r12 | Norway | 11 |
| r12 | Portugal | 25 |
| r12 | Russia | 10 |
| r12 | Scotland | 48 |
| r12 | Singapore | 25 |
| r12 | Spain | 20 |
| r12 | Sweden | 63 |
| r12 | Switzerland | 58 |
| r12 | Thailand | 25 |
| r12 | USA | 4640 |
| r12 | Vietnam | 17 |
| r12 | Wales | 42 |
| r12 | Other | 124 |
| r13 | Australia | 76 |
| r13 | Canada | 29 |
| r13 | England | 57 |
| r13 | France | 42 |
| r13 | Israel | 28 |
| r13 | USA | 220 |
| r13 | Vietnam | 15 |
| r13 | Other | 88 |
| r14 | Other | 2 |
| r15 | Australia | 38 |
| r15 | England | 63 |
| r15 | France | 42 |
| r15 | Israel | 29 |
| r15 | USA | 266 |
| r15 | Other | 63 |
| r16 | Australia | 76 |
| r16 | Canada | 18 |
| r16 | England | 55 |
| r16 | France | 42 |
| r16 | Israel | 28 |
| r16 | USA | 268 |
| r16 | Vietnam | 15 |
| r16 | Other | 88 |

**Supplementary Table 4: Genomes representing each of the major evolutionary lineages represented in our cohort.**

| GISAID ID | Virus name | Collection date | Lineage *(GISAID Clade)* | Originating lab |
| --- | --- | --- | --- | --- |
| EPI_ISL_406801 | Wuhan/WH04/2020 | 2020-01-05 | A (*S*) | General Hospital of Central Theater Command of People's Liberation Army of China |
| EPI_ISL_430131 | USA/WA-S277/2020 | 2020-02-22 | A.1 (*S*) | Seattle Flu Study |
| EPI_ISL_402123 | Wuhan/IPBCAMS-WH-01/2019 | 2019-12-24 | B (*L*) | Institute of Pathogen Biology, Chinese Academy of Medical Sciences & Peking Union Medical College |
| EPI_ISL_412973 | Italy/CDG1/2020 | 2020-02-20 | B.1 (*G*) | Department of Infectious Diseases, Istituto Superiore di Sanità, Roma , Italy |
| EPI_ISL_417922 | Italy/INMI4/2020 | 2020-02-28 | B.1.1 (*GR*) | INMI Lazzaro Spallanzani IRCCS |
| EPI_ISL_426435 | hCoV-19/USA/RI_0882/2020 | 2020-03-09 | B.1.2 (*GH*) | RI State Health Laboratories |
| EPI_ISL_436048 | USA/NY-SURV076/2020 | 2020-03-05 | B.1.3 (*GH*) | NYC Department of Health and Mental Hygiene |
| EPI_ISL_420795 | USA/RI_0556/2020 | 2020-03-01 | B.1.5 (*G*) | RI State Health Laboratory |
| EPI_ISL_420442 | Belgium/BGM-030444/2020 | 2020-03-04 | B.1.12 (*GH*) | KU Leuven, Clinical and Epidemiological Virology |
| EPI_ISL_419811 | Australia/VIC99/2020 | 2020-03-16 | B.1.13 (*GH*) | Victorian Infectious Diseases Reference Laboratory (VIDRL) |
| EPI_ISL_424850 | hCoV-19/USA/IL_0087/2020 | 2020-03-07 | B.1.111 (*GH*) | IL Department of Public Health Chicago Laboratory |
| EPI_ISL_418348 | hCoV-19/Canada/ON_PHL3680/2020 | 2020-03-08 | B.1.114 (*GH*) | Public Health Ontario Laboratories |


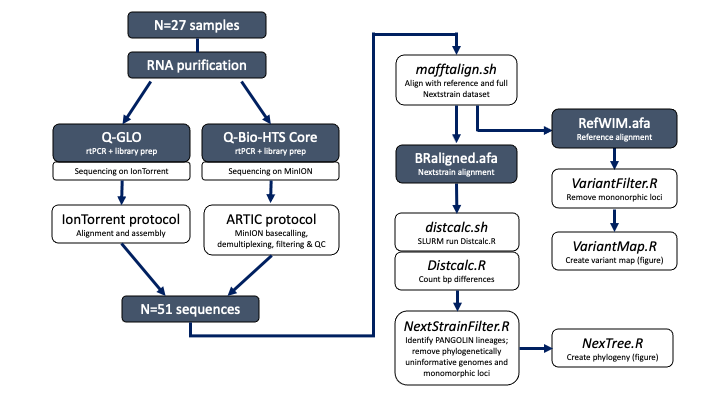


**Supplementary Figure 1. Schematic overview of the bioinformatics pipeline**. Script names are indicated in italics. Further details and updates available at the GitHub repository (https://github.com/ColauttiLab/SARS-CoV_Phylogenomics).

**
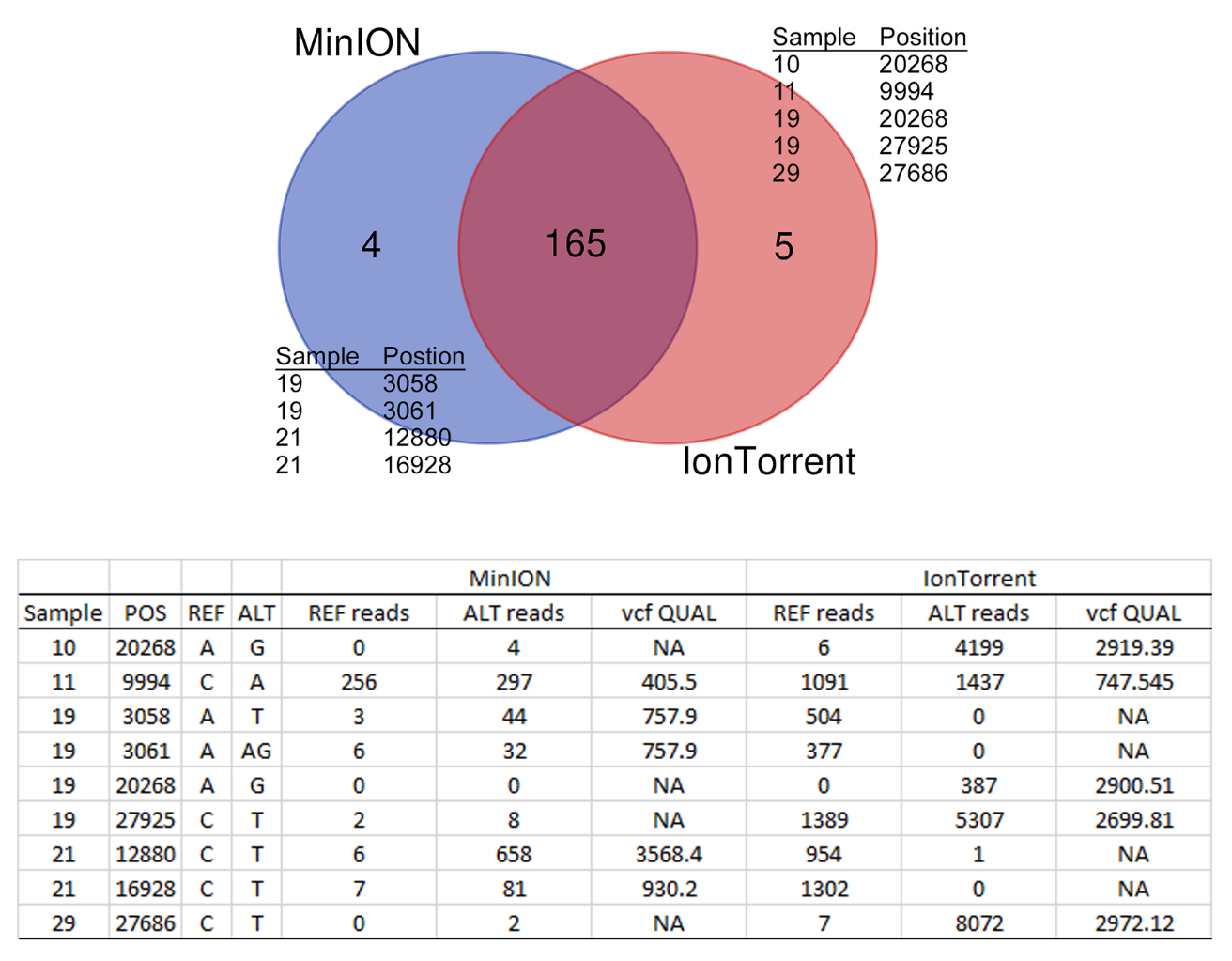
**

**Supplementary Figure 2. Overlap between variants called by the Ion Torrent and MinION sequencing platforms.** One hundred and sixty-five variants were called by both platforms, and only nine variants were called by one but not the other platform. Vcf QUAL is quality score of the variant (-10logP).

**
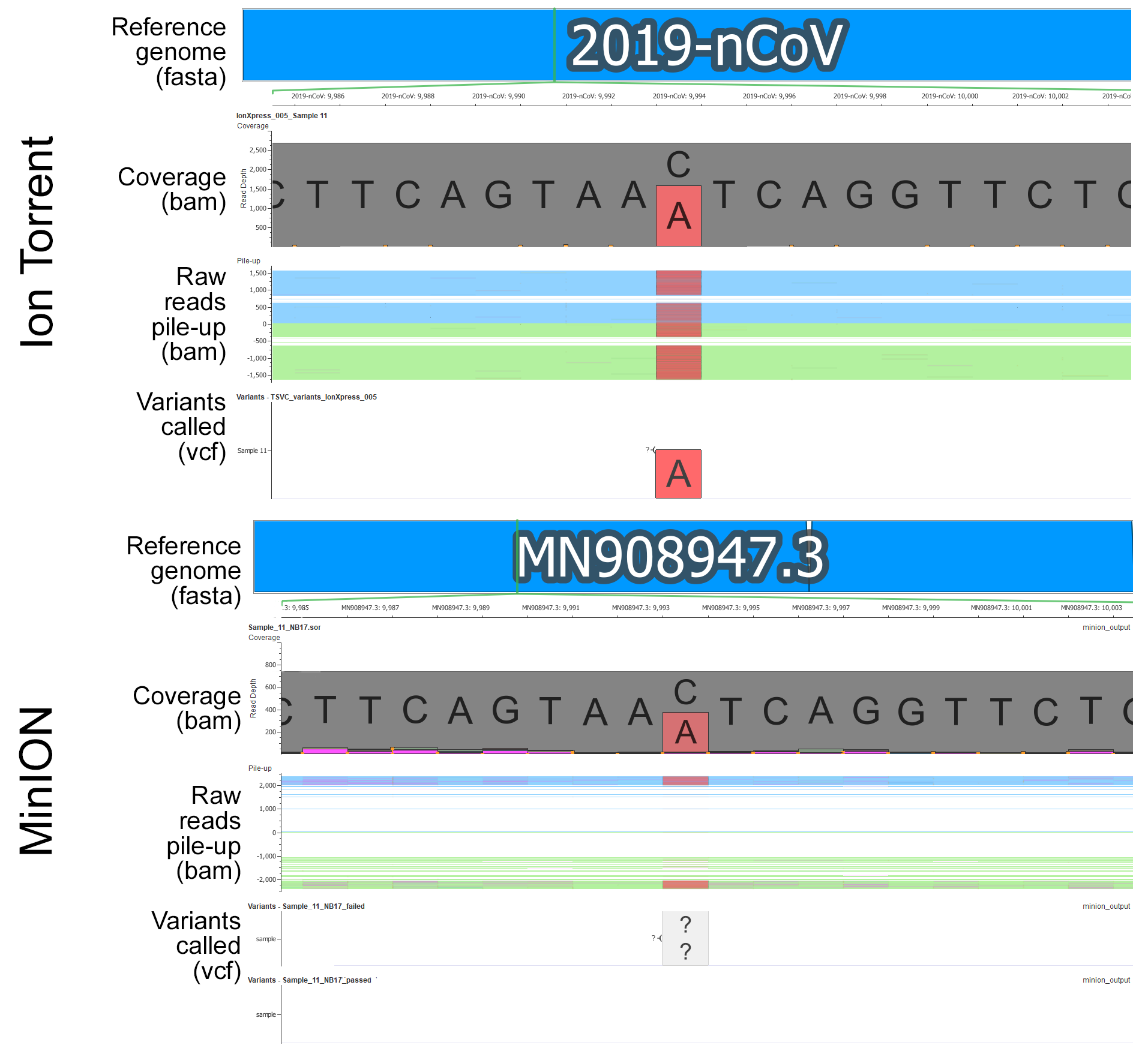
**

**Supplementary Figure 3. Overview of sequencing data from Sample 11 at genome location 9994 viewed in Golden Helix’s Genome Browse.** The Ion Torrent coverage map demonstrates ~2500x coverage at this location of the genome, the variant is present on both forward and reverse reads, and a heterozygous variant called in the vcf file. The MinION coverage map demonstrates ~700x coverage at this location, the variant is present on both forward and reverse reads, and a homozygous variant is called in the fail.vcf file (top) but not the pass.vcf file (bottom).
